# Translation and validation of the Persian version of the caring nurse-patient interaction scale (CNPI-23)

**DOI:** 10.1186/s12912-023-01558-5

**Published:** 2023-10-19

**Authors:** Anis Bahreini, Omsalimeh Roudi RashtAbadi, Saiedeh Haji-Maghsoudi, Roghayeh Mehdipour-Rabori

**Affiliations:** 1 MSc in Nursing, Nursing Research Center, Kerman University of Medical science, Kerman, Iran; 2 Nursing Research Center, Department of Medical-Surgical Nursing, School of Nursing & Midwifery, Kerman University of Medical science, Kerman, Iran; 3 Modeling in Health Research Center, Institute for Futures Studies in Health, Kerman University of Medical Sciences, Kerman, Iran; 4 Department of Biostatistics and Epidemiology, School of Public Health, Kerman University of Medical Sciences, Kerman, Iran; 5 Department of Medical-Surgical Nursing, Razi School of Nursing and Midwifery, Nursing Research Center, Kerman University of Medical Sciences, Haft bagh Alavi Highway, Kerman, Iran

**Keywords:** Nurse-patient Interaction, Caring nurse-patient Interaction Scale, CNPI

## Abstract

**Introduction and objectives:**

Although there is great emphasis on nursing care interaction, there is a lack of knowledge about the quality of nurse-patient care interactions in Iran. The lack of knowledge is mainly related to a lack of short Persian instruments that measure nurse-patient interaction from a caring perspective. The present study aimed to validate a Persian version of the nurse and patient versions of the Caring Nurse-Patient Interaction scale (CNPI-23).

**Methods:**

The scale (CNPI-23) was translated to Persian using the forward-backward translation method. After translation and re-translation, the scale was given to 15 nurses and faculty members of Kerman University of Medical Sciences, and CVI and CVR indices were calculated based on their opinions. The analytical cross-sectional study was conducted in Kerman/Iran in 2022. In this study, 230 working nurses and 230 hospitalized patients in hospitals affiliated with Kerman University of Medical Sciences were recruited using the convenience method to complete the 23-item Caring Nurse-Patient Interaction scale. Exploratory and confirmatory factor analyses were used to analyze the validity of the scale, and Cronbach’s alpha and Raykov’s rho indices were also calculated to evaluate internal consistency and composite reliability. Data were analyzed using R 4-1-2 software.

**Results:**

The scale was completed by 230 nurses and 230 patients. It included four dimensions: humanitarian care, clinical care, comforting care, and communication care. The results of the content validity ratio (CVR) and content validity index (CVI) were acceptable for all items. The minimum value of reliability was 0.49. All the items were approved at the end of the content validity assessment. In the patients’ scale, these four factors explained 81% of the total variance, and for the exploratory model, all the indices show the adequacy of the model. All factor loadings were significant and higher than 0.5. Raycov’s rho and Cronbach’s alpha indices for all numbers were higher than 0.7. The findings of the exploratory factor analysis showed that the nurses’ scale reflected four caring domains, which explained about 62% of the total variance, and the results of Raycov’s rho and Cronbach’s alpha indices confirmed the final fit of this model.

**Conclusion:**

In general, the Persian version of the Caring Nurse-Patient Interaction scale has good validity and reliability and can be used to evaluate the quality of care interaction between Persian-speaking nurses and patients.

## Introduction

Nursing is a process of interpersonal interaction and care provision because every day, nurses encounter different patients and have to meet their varying needs and expectations [1]. Communication is a basic need of patients and a key part of nursing care. Interaction between nurses and patients informs patients about their disease and treatment, and it helps the nurse understand the patient’s anxieties. communication also improves physical, mental, and behavioral outcomes and patient comfort. Most complaints and dissatisfactions in healthcare are due to communication mistakes and lack of effective interaction [2].

Care interaction is one of the most necessary skills in nursing practice, and the correct implementation of nursing interventions requires proper and correct interaction between nurses and other nurses, managers, and patients. Because effective interaction is among the important needs of the patient, it is considered the basis of nurses’ work in caring for patients. The meaning of interaction is mutual respect for professional values and individual abilities using the knowledge and experiences of colleagues through asking for opinions and advice during decision-making [3]. Caring interaction in nursing is how a nurse responds to a patient’s feelings, needs, and information in every care situation. It can address the physical, mental, social, and spiritual aspects of care [4]. Interaction is essential for nursing practice, as it enables nursing to have effective interventions with managers and patients. Interaction also helps nurses make better decisions [5].

The quality of nursing care means providing safe services that satisfy the patient according to nursing standards. Interactions are important sources for evaluating the quality-of-care interaction for the patient and the nurse, but most studies only address the patient’s perspective [6]. Nurse-patient interaction helps patients feel the trust, safety, comfort, confirmation, value, dignity, and well-being that they need. It also affects their hope, anxiety, and depression. It can balance the patient’s life and reduce their suffering [7]. Nurses can use assessment tools to measure their care competence.

Jean Watson’s theory emphasizes how nurses care for patients, how care affects recovery, and how care enhances health. She describes care in nursing as a scientific, ethical, esthetic and professional process that involves physical, mental, psychological, and socio-cultural interactions between two people. She guides nurses to maintain a caring interaction with love, respect, and trust. Her goal is to help nurses create a treatment setting that meets the patients’ needs. Her theory of human care enables nurses to improve the nurse-patient relationship [8]. She listed ten factors for patients care. These 10 factors are [1] humanistic-altruistic value system [2], faith-hope [3], sensitivity to self and others [4], helping-trust in human care relationships [5], expression of positive and negative emotions [6], creative problem-solving in the caring processes [7], transpersonal teaching-learning [8], supportive, protective, and/or corrective mental, physical, societal and spiritual environment [9], human needs assistance, and [10] existential-phenomenological-spiritual forces [9].

The Caring Nurse-Patient Interaction-Long Scale was created by Cossette et al. They created it to evaluate attitudes and behaviors connected to Watson’s 10 factors. They made a shorter version (CNPI-Short Scale) because the 70-item questionnaire was too long for clinical research, especially with very ill patients. The shorter scale has three caring domains that come from the original 10 factors [10].

Some studies have used and psychometrically tested this scale in different languages. For instance, Calong et al. validated the scale among Filipino nurses [11], and Sharour translated the patient version of the scale into Arabic and validated it [3]. In China, Ma et al. developed and validated this scale from the nurses’ perspective [12]. Although these countries are in Asia, Persian is not the language that people use in these countries. Additionally, the culture of these countries is completely different from the Iranian culture, and some of these articles validated the scale only from the perspective of either patients or nurses. Consequently, the lack of articles that validated the scale in Persian compelled the researchers to undertake the translation and validation of the Caring Nurse-Patient Interaction for both nurses and patients in Persian.

## Method

### Design

The present study is an analytical cross-sectional study with validation and psychometric testing conducted in 2022 in Iran.

### Setting

The study was done in Kerman, Iran. Kerman is the largest province in Iran. The study recruited participants from three major hospitals affiliated with Kerman University of Medical Sciences.

### Sampling

The statistical population of this study consisted of 230 working nurses and 230 patients hospitalized in hospitals affiliated with Kerman University of Medical Sciences in 2022. The researchers selected the patients and the nurses randomly by checklist.

### Inclusion/exclusion criteria

Inclusion criteria for nurses included having at least six months of clinical work experience and enough time to complete the scale.

Inclusion criteria for patients included being hospitalized in the heart or general departments for at least three days, having a good general condition, reading and writing literacy in the Persian language, and consent to participate in the study.

Exclusion criteria included failure to complete the scale (more than 10%) and having dementia or psychological problems according to self-report.

### Study instruments

The researchers translated and validated the Caring Nurse-Patient Interaction Scale (CNPI-23), This scale consists of 23 items rated using a five-point Likert scale from 1 (never) to 5 (always), which reflects four caring domains: humanistic care (questions 1 to 4), relational care (questions 5 to 11), clinical care (questions 12 to 20), and comforting care (questions 21 to 23). In the present research, two separate scales were used to examine the caring interaction from the patient’s and nurse’s perspectives, the patient’s scale from the patient’s point of view and the nurses’ scale from the nurses’ point of view, but the options of both scales were the same. Each question is stated positively, and the scores range from 23 to 115, with a minimum score of 23 and a maximum score of 115. A higher score indicates a higher quantity and quality of interaction between the nurse and patient [4].

### Validity and reliability

In the first step, the English version of the Caring Nurse-Patient Interaction scale was translated to Persian. The researchers of this study obtained permission from the author of the scale through email, and then the English scale was translated through forward-backward translation. At first, the researcher, whose native language was Persian and who had sufficient knowledge about the concepts raised in the scale, translated the English scale to Persian. It should be noted that more attention has been paid to the meaning of the translations for the patients than verbal similarity with the original (the literal translation was not used). In the second stage, two faculty members of Kerman Razi Midwifery and Nursing Faculty who were familiar with Persian and English reviewed the instrument translation and discussed the inconsistencies between the original and the translated version, applied the necessary corrections, and translated it into English. In the next stage, the translation was checked by 15 faculty members of Kerman University of Medical Sciences and nurses with a master’s degrees who were fluent in Persian and English for content validity and compatibility with Iranian culture. Then, the final corrections were made, and the scale was compiled.

The statistical population of this study consisted of 230 working nurses and 230 patients. For each item, 10 people were recruited to complete the caring nurse-patient interaction scale. These samples were used for exploratory and confirmatory analysis. The researchers selected nurses and patients randomly. First, the researchers explained the aim of the study to them, and then the participants completed the scale.

The psychometric properties of the scale were evaluated using the content validity index and the content validity ratio. A form including an explanation of the study topic and the objectives was prepared to check the content validity. Then, the experts, including 15 faculty members of Razi School of Nursing and Midwifery and nurses with master’s degrees were asked to examine each area based on a three-part spectrum from the point of view of necessity (the item is necessary, “he item is useful but not necessary, or “the item is not necessary), and then the CVR was calculated. Also, the experts panel evaluated the CVI of each item using the three criteria of simplicity (difficult, simple, or quite simple), clarity (not clear, clear, or quite clear), and relevance (not relevant, requires serious revision, relevant but needs to be revised, or completely relevant). After collecting the experts’ opinions, the necessary corrections were made to the scale. The CVI of each item was calculated as the number of respondents giving a 3 or 4 rating divided by the total number of respondents. The validated scale was administered among the patients and nurses of selected hospitals of the Kerman University of Medical Sciences.

### Analysis

Data were analyzed using R 4-1-2 software. *P*-values less than 0.05 were considered significant. Univariate normality was assessed through the Anderson-Darling test, and multivariate normality was evaluated using the Henze-Zirkler’s test. Exploratory factor analysis (EFA) and confirmatory factor analysis (CFA) was used for construct validity analysis for both groups of nurses and patients separately. Cronbach’s alpha and Raykov’s rho indices were calculated to evaluate internal consistency and composite reliability. The polychoric correlation coefficient matrix was used in exploratory factor analysis and varimax rotation was used to determine the dimensions. The KMO index was calculated and Bartlett’s *P*-value was also calculated to evaluate the sufficiency of the data for exploratory factor analysis. The number of factors was examined using the scree plot. Confirmatory factor analysis was evaluated using the robust maximum likelihood method. Indices like X^2^/df, comparative fit index (CFI), Tucker-Lewis index (TLI), root-mean-square error (RMSE), and standardized root mean square residual (SRMR) were also used to determine the model’s appropriate fit.

## Findings

Among the 230 patients and 230 nurses participating in this study, the average age of patients was 54.3 years old, and the average age of nurses was 37.22 years old. The number of participating female patients and nurses was slightly higher than males (53.5% and 51.7%, respectively). The awareness level of patient communication methods among nurses was 66.5%, which is average, and 69.6% of nurses had completed patient communication or patient training courses. Among the patients, 36.5% had more than one underlying disease, and in about 40% of them, the disease started or was diagnosed about a year ago (Table 1).

The results of the reliability ratio (CVR) and content validity index (CVI) were also evaluated, according to Lavshe’s table. The minimum value of the reliability ratio for 15 experts was 0.49, which was acceptable for all items. At the end of the content validity check, all the items were approved.

**Table 1:** Demographic characteristics of patients and nurses

Variable		Frequency	Percentage
patients			
Gender	Male	107	46.5
	Female	123	53.5
Marital status	Married	196	85.2
	Single	34	14.8
Insurance Status	Yes	210	91.3
	No	20	8.7
Job	Self-employed	61	26.5
	Employee	44	19.1
	Retired	29	12.6
	Homemaker	72	31.3
	Unemployed	24	10.4
Education	Illiterate and Primary education	77	33.5
	Middle school and high school	85	37.0
	Higher education	68	29.5
Underlying disease	No disease	47	20.4
	Heart disease	28	12.2
	Lung disease	24	10.4
	Diabetes	25	10.9
	Other diseases	45	19.6
	Several underlying diseases	61	26.5
The time of onset of the disease	Less than 1 year	92	40.0
	1 to 5 years	74	32.2
	5 to 10 years	41	17.8
	More than 10 years	22	9.6
Monthly income	Less than 3 million tomans	97	42.3
	3 to 6 million tomans	46	20.0
	6 to 9 million tomans	74	32.2
	More than 9 million tomans	12	5.2
Nurses			
Gender	Male	119	51.7
	Female	111	48.3
Marital status	Married	160	69.6
	Single	70	30.4
Education	Bachelor’s degree	195	84.8
	Master’s degree	35	15.2
The level of awareness of methods of communication with the patient	Low	52	22.6
	medium	153	66.5
	High	25	10.9
Passing courses on communication with patients or teaching patients	Yes	160	69.6
	No	70	30.4

**Fig. 1:**
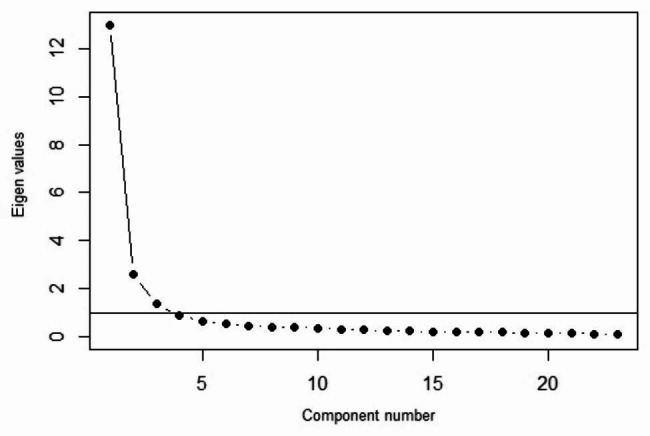
Scree plot of exploratory factor analysis for the patients’ scale

In the exploratory factor analysis, the KMO index and Bartlett’s *P*-value for both scales showed that the exploratory factor analysis was suitable for the data (Table 2). The number of factors was considered to be four. The results of the exploratory factor analysis of the patients’ scale are presented in Table 3. Based on the factorial loads of the items (Table 3) and scree plot (Fig. 1), four domains were tested in this analysis. In the exploratory structure of the patient’s scale, the last two dimensions of the original version of the scale (comforting and humanistic care dimensions) were merged into one dimension. Questions 1 to 5 (clinical care dimension), 6 to 9 (relational care dimension), 10 to 16 ( humanistic care dimension), and 17 to 23 (comforting care dimension) corresponded to the different dimensions of the patients’ scale, which explained 81% of the total variance.

**Table 2:** KMO sampling adequacy index and Bartlett’s sphericity test results

		Patients’ scale	Nurses’ scale
Sampling Adequacy Index (KMO)		0.86	0.83
Bartlett’s sphericity test	Chi-square statistic	7672.64	4031.45
	Degrees of freedom	253	253
	*P*-value	< 0.001	< 0.001

**Table 3:** The results of exploratory factor analysis for the patients’ scale

Dimensions	Items	The first factor	The second factor	The third factor	The fourth factor
Clinical care	1- The nurse knows how to perform care measures (for example, intravenous injection, dressing, etc.).	0.180	**0.789**	0.399	0.059
	2- The nurse knows how to work with specialized equipment (for example, working with infusion pumps, monitors, etc.).	0.168	**0.841**	0.263	0.112
	3- The nurse examines the effect of prescription drugs on the improvement of symptoms (for example, nausea, pain, constipation, anxiety, etc.).	0.143	**0.759**	0.256	0.129
	4- The nurse teaches me the methods of prevention as well as eliminating the side effects of drugs or treatment methods.	0.224	**0.767**	0.212	0.240
	5- The nurse knows what to do in situations where they must act quickly.	0.255	**0.755**	0.249	0.309
Relational care	6- The nurse helps me in care that I cannot do by myself.	0.342	0.533	0.330	**0.553**
	7- The nurse showed their ability and skill in interacting with me.	0.395	0.420	0.395	**0.644**
	8- The nurse closely examined my health condition (performed nursing care).	0.422	0.469	0.340	**0.527**
	9- The nurse gave me the opportunity to take care of myself.	0.32	0.498	0.374	**0.516**
Humanistic care	10- The nurse helped me to be more careful about my health in my life.	**0.735**	0.240	0.278	0.300
	11- The nurse helped me to discover the importance of health in life.	**0.856**	0.1668	0.191	0.234
	12- The nurse helped me to choose what I want my relatives to bring me.	**0.863**	0.194	0.258	0.160
	13- The nurse helped me to discover the meaning I gave to my health condition	**0.920**	0.186	0.181	0.119
	14- The nurse helped me to identify effective solutions to problems.	**0.860**	0.175	0.304	0.132
	15- The nurse helped me to look at health-related issues from other angles.	**0.829**	0.0227	0.343	0.038
	16- The nurse helped me to try to identify the consequences of behaviors that affect my health.	**0.810**	0.195	0.334	0.231
Comforting care	17- By considering me as a complete person, the nurse showed that they are interested in solving my health problem.	0.493	0.321	**0.579**	0.217
	18- The nurse gave me hope whenever they could.	0.406	0.255	**0.619**	0.354
	19- The nurse cared about my efforts in self-care and recovery	0.401	0.238	**0.631**	0.352
	20- The nurse accepted me and did not reject me.	0.369	0.294	**0.681**	0.189
	21- The nurse respected my privacy (for example, they did not expose me to others for no reason).	0.310	0.381	**0.757**	0.0115
	22- The nurse considered my basic needs (e.g. sleeping, hygiene, etc.).	0.258	0.382	**0.775**	0.183
	23- The nurse performed treatment and gave me medications according to the schedule.	0.286	0.389	**0.811**	0.122
Expressed cumulative variance		0.289	0.508	0.719	**0.810**

**Fig. 2:**
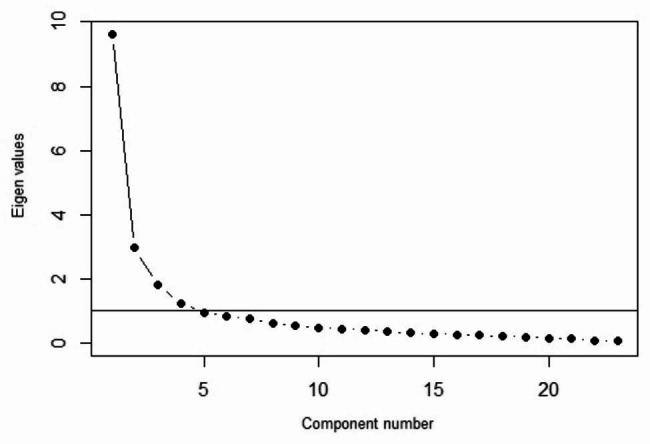
Scree plot of exploratory factor analysis for the nurses’ scale

The results of the exploratory factor analysis of the nurses’ scale are presented in Table 4. The KMO index and the results of Bartlett’s sphericity test confirmed the appropriateness of the analysis (Table 2). Four domains were tested considering the factorial loads of the items (Table 2) and scree plot (Fig. 2), and they explained about 62% of the total variance. In the scale, the first dimension (clinical care) was divided into two dimensions and the last two dimensions (comforting and humanistic care) were merged together. Questions 1 to 3 (the clinical care dimension), 4 to 9 (the relational care dimension), 10 to 16 (the humanistic care dimension), and 17 to 23 (the comforting care dimension) correspond to the different dimensions of nurses’ scale (Table 4).

It was noted that a few items exhibited cross-loadings when a factor loading cut-off value of 0.32 was used. However, these items were not removed.

**Table 4:** The results of exploratory factor analysis for nurses

Dimensions	Items	The first factor	The second factor	The third factor	The fourth factor
Clinical care	1- I know how to perform care measures (for example, intravenous injection, dressing, etc.).	**0.760**	0.330	0.057	0.227
	2- I know how to work with specialized equipment (for example, infusion pumps, monitors, etc.).	**0.710**	0.333	0.198	0.052
	3- I know how to check the effect of prescription drugs for the patient (for example, nausea, pain, constipation, anxiety, etc.).	**0.649**	0.494	0.212	0.246
Relational care	4- I know how to teach the patient how to prevent and eliminate the side effects of drugs or treatment methods.	0.327	**0.833**	0.164	0.155
	5- I know what to do in situations where I have to act quickly.	0.240	**0.59**	0.152	0.263
	6- I know how I should help the person in care activities they are not able to do on their own.	0.249	**0.669**	0.168	0.273
	7- I know how to show my ability and skill in interacting with the patient.	0.147	**0.761**	0.222	0.219
	8- I know how to closely examine the health status of the patient.	0.192	**0.722**	0.222	0.261
	9- I know how to give the patient the opportunity to practice self-care.	0.262	**0.619**	0.286	0.221
Humanistic care	10- I know how to help the client to be more careful about their health.	0.200	0.192	**0.609**	0.140
	11- I know how to help the client discover what is important in their life related to health.	0.056	0.235	**0.722**	0.145
	12- I know how to help the client to choose what they want their relatives to bring them.	0.099	0.006	**0.724**	0.016
	13- I know how to help the client to discover the meaning they have given to their health condition.	0.134	0.124	**0.732**	0.002
	14- I know how to help the client to identify how to reduce their problems.	0.053	0.147	**0.680**	0.206
	15- I know how to help the client to look at issues from other angles.	-0.20	0.175	**0.754**	0.063
	16- I know how to identify the consequences of the client’s behavior together with them.	0.034	0.186	**0.658**	0.249
Comforting care	17- I know how to consider the client as a complete person and show that I care and am interested in solving their health problem.	-0.019	0.367	0.407	**0.424**
	18- I know how to encourage the client to be hopeful whenever possible.	0.024	0.422	0.368	**0.506**
	19- I care about the patient’s efforts in self-care and recovery.	-0.032	0.263	0.265	**0.672**
	20- I accept others as they are.	-0.036	0.0155	0.199	**0.687**
	21- I know how to respect the client’s privacy (for example, not expose them to others for no reason).	0.343	0.136	0.035	**0.600**
	22- I know how to consider the client’s basic needs (for example, sleeping, hygiene, etc.).	0.316	0.308	0.066	**0.598**
	23- I know how to carry out treatment measures and prescribe drugs according to the planned schedule.	0.375	0.215	-0.009	**0.746**
Expressed cumulative variance		0.100	0.292	0.475	**0.615**

According to the literature review, df/x2 less than 3, CFI and TLI greater than 0.9, RMSE less than 0.08, and SRMR less than 0.05 indicate the good fit of the model. Confirmatory factor analysis showed good fit for both scales (Table 5).

**Table 5:** Indices related to confirmatory factor analysis of the scales

scale	χ^2^/df	*P*-value	Robust Comparative Fit Index (CFI)	Robust Tucker-Lewis Index (TLI)	Robust root mean square error of approximation (RMSEA) (90% CI)	Standardized Root Mean Square Residual (SRMR)
Patients’ questionnaire	1.468	< 0.001	0.975	0.970	0.051 (0.038–0.063)	0.047
Nurses’ questionnaire	1.217	0.017	0.977	0.973	0.032 (0.015–0.045)	0.057

**Fig. 3:**
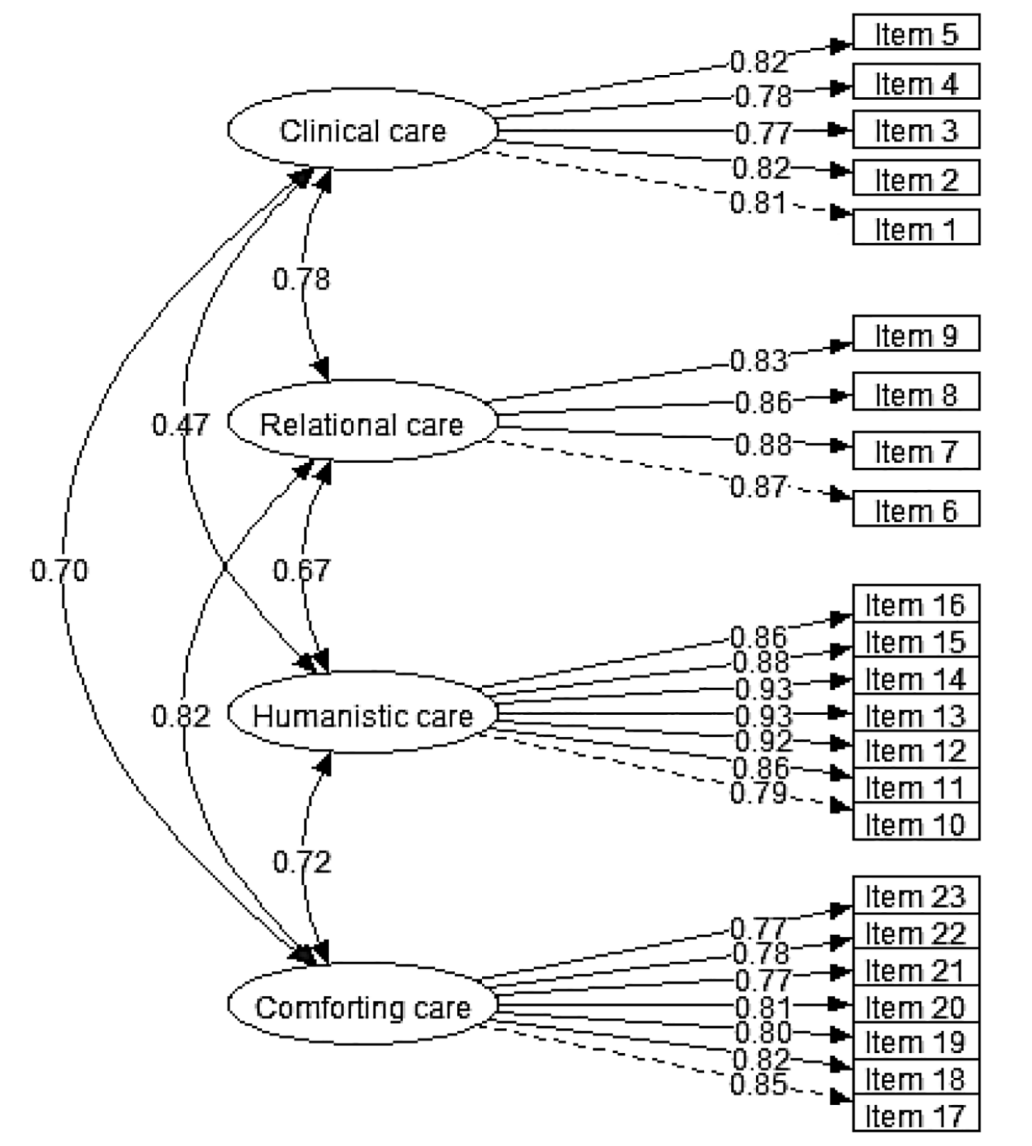
The results of confirmatory factor analysis of the patients’ scale

For the confirmatory model of patients, all indicators show the adequacy of the model, and all factor loadings were significant. Raycov’s rho and Cronbach’s alpha indices were also greater than 0.70. The results of Raycov’s rho and Cronbach’s alpha indices confirm the final fit of this model (Table 6; Fig. 3).

**Table 6:** Results of confirmatory factor analysis and reliability indices of the patients’ scale

Dimension	Item	Estimate	SE	Standardized Estimate	*P*-value	Raykov’s rho	Cronbach’s alpha
Clinical care	1	1.00		0.81	< 0.001	0.90	0.89
	2	1.25	0.07	0.82	< 0.001		
	3	1.19	0.08	0.77	< 0.001		
	4	1.29	0.13	0.78	< 0.001		
	5	1.26	0.12	0.82	< 0.001		
Relational care	6	1.00		0.87	< 0.001	0.92	0.92
	7	1.00	0.04	0.88	< 0.001		
	8	1.00	0.06	0.86	< 0.001		
	9	0.98	0.05	0.83	< 0.001		
Humanistic care	10	1.00		0.79	< 0.001	0.96	0.96
	11	1.20	0.06	0.86	< 0.001		
	12	1.33	0.07	0.92	< 0.001		
	13	1.37	0.08	0.93	< 0.001		
	14	1.35	0.08	0.93	< 0.001		
	15	1.28	0.08	0.88	< 0.001		
	16	1.28	0.07	0.86	< 0.001		
Comforting care	17	1.00		0.85	< 0.001	0.93	0.89
	18	0.94	0.06	0.82	< 0.001		
	19	0.90	0.06	0.80	< 0.001		
	20	0.80	0.05	0.81	< 0.001		
	21	0.76	0.07	0.77	< 0.001		
	22	0.81	0.07	0.78	< 0.001		
	23	0.76	0.07	0.77	< 0.001		

**Fig. 4:**
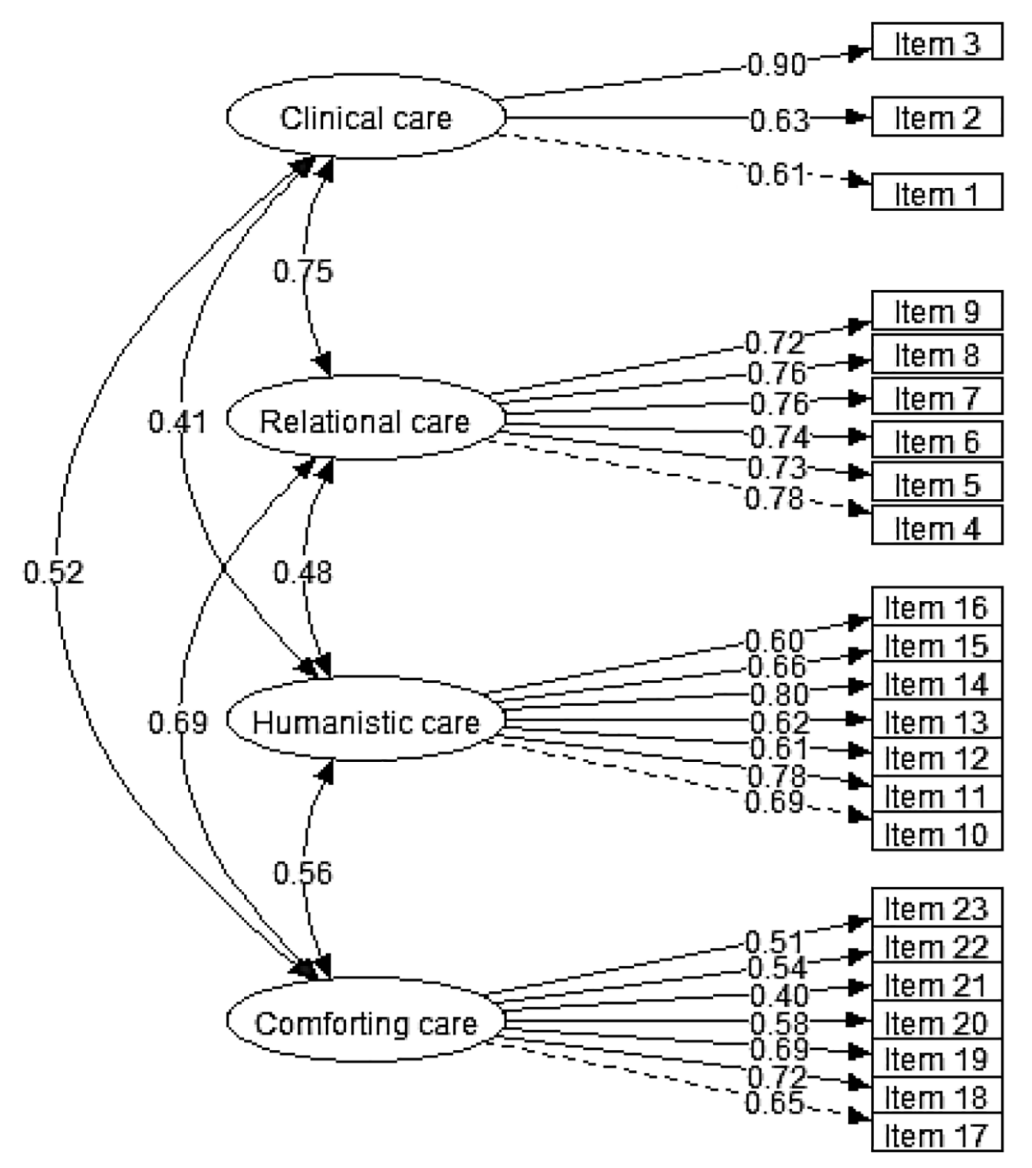
The results of confirmatory factor analysis of the nurses’ scale

For the confirmatory model of nurses, all indicators show the adequacy of the model (Table 5), all factor loadings were also significant. All standard coefficients were greater than 0.40, and Raykov’s rho index and Cronbach’s alpha coefficient were greater than 0.70 (Table 7; Fig. 4).

**Table 7:** The results of confirmatory factor analysis and reliability indices of the nurses’ scale

Dimension	Item	Estimate	SE	Standardized Estimate	*P*-value	Raykov’s rho	Cronbach’s alpha
Clinical care	1	1.00		0.61	< 0.001	0.74	0.79
	2	1.28	0.16	0.63	< 0.001		
	3	1.89	0.24	0.90	< 0.001		
Relational care	4	1.00		0.78	< 0.001	0.89	0.88
	5	0.88	0.07	0.73	< 0.001		
	6	0.97	0.07	0.74	< 0.001		
	7	1.04	0.09	0.76	< 0.001		
	8	1.00	0.09	0.76	< 0.001		
	9	0.98	0.10	0.72	< 0.001		
Humanistic care	10	1.00		0.69	< 0.001	0.85	0.86
	11	1.16	0.09	0.78	< 0.001		
	12	0.92	0.09	0.61	< 0.001		
	13	0.88	0.10	0.62	< 0.001		
	14	1.12	0.10	0.80	< 0.001		
	15	0.96	0.11	0.66	< 0.001		
	16	0.86	0.12	0.60	< 0.001		
Comforting care	17	1		0.65	< 0.001	0.76	0.80
	18	1.12	0.10	0.73	< 0.001		
	19	1.01	0.12	0.69	< 0.001		
	20	0.78	0.12	0.57	< 0.001		
	21	0.50	0.11	0.40	< 0.001		
	22	0.66	0.12	0.54	< 0.001		
	23	0.57	0.10	0.51	< 0.001		

## Discussion

The study aimed to validate a Persian version of the nurse and patient versions of the Caring Nurse-Patient Interaction scale (CNPI-23). The present study indicated the Persian version of the Caring Nurse-Patient Interaction scale has good validity and reliability and can be used to evaluate the quality of the care interaction between Persian-speaking nurses and patients.

Access to valid and reliable instruments for measuring caring nurse-patient interaction is considered the first requirement for planning and measures related to maintaining and improving the quality of nursing care [13]. Due to the lack of scientific and native instruments to collect information to measure the caring nurse-patient interaction in Iran, the current scale was translated into Persian and validated.

The CNPI-23 is commonly used for measuring the caring nurse-patient interaction. The scale has been used in many countries [14–16]. In Spain and Poland, the CNPI-70 scale (long version) has been translated into Spanish and Polish, respectively, and tested [17, 18].

The results of the content validity in present study showed that the process used in translating the scale into Persian was correct and logical and the Persian content is not only consistent with the original version but also clear and expressive for the target population. Repeatability is the reliability of a test, which is measured by different methods [18].

The internal stability of the instrument method was used to assess the reliability of the scale. Cronbach’s alpha coefficient was used for the whole scale and also for each of the dimensions separately. Cronbach’s alpha coefficient for all factors in the present study ranged from 0.73 to 0.96. In a study by Kathyrine Calong and Gil Platon Soriano (2019) in the Philippines, the internal correlation of the instrument for all sub-dimensions was reported between 0.81 and 0.94, and in this study also the instrument had good internal consistency [11]. By comparing the validity and reliability values obtained from this study and other studies conducted in this field, it seems that these values are close to each other and have an acceptable match.

In the present study, exploratory and confirmatory factor analysis was used to investigate the structure of the factors. Factor analysis with different factor rotation methods extracted four factors that explain about 62% of the variance. These factors, as in the original version, include clinical care, relational care, humanistic care, and comforting care [20]. The factorial structure of the scale designed by Cossette et al. was also investigated in the Philippines and China. The results of the factorial structure of the present study are similar to the original version of the scale designed by Cossette et al. (2008), and they are also similar to the studies by Kathyrine Calong Calong and Gil Platon Soriano in the Philippines and Hui-Chun Chung et al. in China [11, 19, 20].

Colang et al., using a sample consisting of 124 subjects in Manila, reported that the Caring Nurse-Patient Interaction scale developed by Cossette et al. is a valid and reliable instrument [4]. Chung et al. also tested the validity and reliability of this scale on 365 nurses in China and reported that the reliability and validity coefficients of the factors are high [19]. These findings mean that this scale has sufficient accuracy and reliability in measurement and the questions raised in the scale are a good representation of the wide range of caring nurse-patient interactions.

The strengths of the current study include the use of 10 participants per item. However, the generalizability of the findings of this research may be limited because the samples were selected only from patients who were hospitalized in heart and general wards and nurses who worked in Kerman hospitals. Therefore, to confirm the results, it is suggested that research be conducted on larger samples and nurses working in other parts of Iran in the future. Also, as in this research, a questionnaire was used to collect data, some people may have refused to provide real answers and given unrealistic answers.

## Conclusion

The Caring Nurse-Patient Interaction scale (CNPI-23) has good validity and reliability to evaluate the caring nurse-patient interaction and can be used to measure the caring nurse-patient interaction in all research and treatment departments. The use of the scale in future studies is recommended to researchers interested in this field.

CNPI-23 can be used in all nursing fields to improve the quality-of-care interactions. Nursing managers can also use this scale to check the quality-of-care interactions and provide solutions for improvement.

## Data Availability

The data are available upon request from the corresponding author after signing the appropriate documents in line with ethical applications and the decision of the Ethics Committee.
